# Synergistic Antimicrobial Activity of BrSPR20-P1 Peptide and Silver Nanoparticles Against Pathogenic Bacteria

**DOI:** 10.3390/ijms26167832

**Published:** 2025-08-13

**Authors:** Thanyamai Thongin, Somchai Sawatdee, Nuttapon Songnaka, Jumpei Uchiyama, Theanchai Wiwasuku, Teerapol Srichana, Titpawan Nakpheng, Apichart Atipairin

**Affiliations:** 1 School of Pharmacy, Walailak University, Nakhon Si Thammarat 80161, Thailand; thanyamai.th@mail.wu.ac.th (T.T.); somchai.sa@wu.ac.th (S.S.); nuttapon.so@wu.ac.th (N.S.); 2 Drug and Cosmetic Excellence Center, Walailak University, Nakhon Si Thammarat 80161, Thailand; 3 Department of Bacteriology, Graduate School of Medicine, Dentistry, and Pharmaceutical Sciences, Okayama University, Okayama 700-8558, Japan; uchiyama@okayama-u.ac.jp; 4 School of Science, Walailak University, Thasala, Nakhon Si Thammarat 80161, Thailand; theanchai.wi@wu.ac.th; 5 Drug Delivery System Excellence Center and Department of Pharmaceutical Technology, Faculty of Pharmaceutical Sciences, Prince of Songkla University, Hat Yai 90112, Thailand; teerapol.s@psu.ac.th (T.S.); titpawan.n@psu.ac.th (T.N.)

**Keywords:** antimicrobial peptide, *Brevibacillus* sp. SPR20, silver nanoparticle, synergistic effect

## Abstract

Bacterial infection is a cause of life-threatening diseases. The emergence of antimicrobial-resistant bacteria exacerbates this situation, highlighting the need for the discovery of new antimicrobial agents. Our previous study identified a novel antimicrobial peptide, BrSPR20-P1 (P1), which showed potential activity against MRSA. Additionally, silver nanoparticles (AgNPs) exhibit broad-spectrum antibacterial activity, capable of killing multidrug-resistant bacteria. The combination of antimicrobial agents presents a novel strategy for combating these pathogens. This study aimed to evaluate the antibacterial activity of the combination of P1 and AgNPs. It revealed that the combinations showed synergy. The P1 and AgNP mixture at a concentration of 1 and 8 µg/mL (1:8) doubled the activity against *S*. *aureus* and MRSA, while that combination of 64 and 64 µg/mL (64:64) exhibited broad-spectrum activity, expanding to *E*. *coli* with a 32-fold increase. These combinations exhibited a bactericidal effect, showing the rapid killing of tested bacteria at 10× MIC, with killing rates during the first 3 h ranging from 4.04 ± 0.01 to 4.31 ± 0.03 h^−1^. The P1 and AgNP mixtures caused a low risk of antibacterial resistance up to 30 passages. It was demonstrated that the synergistic activity of P1 and AgNPs occurred through the disruption of cell walls and membranes, leakage of intracellular materials, and cell lysis. Additionally, the mixtures appeared to interact with bacterial genomic DNA, as indicated by a gel retardation assay. These activities of the combinations were concentration-dependent. The 1:8 µg/mL mixture caused low hemolysis and cytotoxicity and did not impede the wound healing process. In contrast, although the 64:64 µg/mL mixture showed excellent antibacterial efficacy, it was toxic to erythrocytes and mammalian cells. It implies that dose optimization is required to balance its efficacy and toxicity. Therefore, the P1 and AgNP combinations exhibit synergistic antimicrobial activity and have the potential to resolve bacterial infections.

## 1. Introduction

Bacterial infection is a public health problem worldwide with an estimated 13.7 million deaths in 2019 [1]. *Staphylococcus aureus* caused the highest number of bacteria-related mortalities, followed by *Escherichia coli*, *Streptococcus pneumoniae*, *Klebsiella pneumoniae*, and *Pseudomonas aeruginosa*, each responsible for more than 500,000 deaths. *S*. *aureus* and *E. coli* were the harmful pathogens associated with the leading causes of fetal bloodstream and abdominal infections, respectively [1]. The emergence of drug-resistant bacteria exacerbated the infectious issue, making pathogens challenging to treat and resulting in higher mortality rates. Methicillin-resistant *S*. *aureus* (MRSA) was the most threatening bacterium associated with 550,000 deaths, while fluoroquinolone-resistant bacteria (*E. coli* and *Acinetobacter baumannii*) individually affected more than 30,000 fatalities [2]. Bacteria acquire a broad range of resistance mechanisms that render most antibiotics ineffective. These include reduced membrane permeability, enhanced efflux pumps, change in drug target sites, modification or degradation of antimicrobial agents, and adaptive responses such as biofilm development [3]. The World Health Organization (WHO) recently announced the bacterial priority pathogens list in 2024 to direct research and development initiatives for new antimicrobial drugs, make public health interventions, and conduct antimicrobial resistance surveillance [4]. MRSA was in the high-priority group, whereas Enterobacterales, such as *E. coli*, resistant to carbapenems or third-generation cephalosporins, were listed as the critical-priority pathogens. A study revealed that *S. aureus* and *E. coli* were responsible for almost 50% of the deadly burden of antimicrobial resistance in high-income countries [1]. It requires the development of novel antimicrobial agents or the establishment of health policies to overcome such situations.

Antimicrobial peptides (AMPs) are small molecules, generally comprising 12 to 50 amino acids with a high proportion of cationic and hydrophobic residues. AMPs have a broad spectrum of antimicrobial activity, inhibiting or eradicating pathogens (bacteria, fungi, viruses, and cancerous cells) [5]. Typically, AMPs attach to microbial surfaces by binding their positive charges to negatively charged microbial membranes via electrostatic interactions. The hydrophobic domains of AMPs facilitate their integration into the cell membrane, resulting in membrane damage and cell lysis. Moreover, they can penetrate cell membranes to interfere with cellular components, ultimately resulting in cell death [6]. Therefore, AMPs affect multiple targets and provide advantages over traditional antibiotics in tackling drug-resistant bacterial infections. For example, several peptide antibiotics (bacitracin, gramicidin S, and polymyxin B) have been approved for clinical application. They have net positive charges at physiological pH and bind to the bacterial membrane, leading to the disruption of the membrane integrity and then cell death [7]. In our previous investigation, we isolated *Brevibacillus* sp. SPR20, which produced several AMPs, killing Gram-positive strains (*S. aureus* and MRSA) [8]. The BrSPR20-P1 peptide (P1) was the most potent anti-MRSA substance, exhibiting a minimum inhibitory concentration (MIC) of 2 µg/mL, comparable to vancomycin [9]. This peptide comprises 15 residues (VVVNVLVKVLPPPVV) and contains an amphipathic character. The antibacterial action of P1 includes membrane disruption, release of intracellular materials, and cell lysis.

Recent advancements in nanotechnology have made silver nanoparticles (AgNPs) more attractive. AgNPs have a size range of 1–100 nm, making them have a high surface area and exhibiting distinctive characteristics [10]. They have demonstrated antimicrobial efficacy against pathogenic microorganisms, including multidrug-resistant bacteria. The exact mechanism by which AgNPs possess antibacterial activity is not well understood. However, it is postulated that AgNPs accumulate in the bacterial membrane, causing membrane instability, leakage of intracellular contents, and subsequently cell death. Moreover, AgNPs can interact with intracellular molecules, such as deoxyribonucleic acid (DNA) and proteins. Numerous studies have shown that AgNPs alter enzymes in the respiratory chain, increasing the production of reactive radical species, which destroy cell structures and initiate the apoptotic process. The oxidative release of silver ions from AgNPs also binds to biomolecules, inducing DNA damage and altering metabolic pathways [11,12].

The combination treatment has gained significant attention because it targets various bacterial components, resulting in a broad spectrum of antibacterial activity, especially against multidrug-resistant strains [13]. Moreover, this strategy can reduce the dose, thereby mitigating the risk of drug toxicity. The concurrent use of AMPs and AgNPs may exhibit synergistic, antagonistic, or additive effects on pathogenic bacteria. For instance, the combination of bacitracin and AgNPs showed significantly improved antibacterial activity against *E. coli* and *P. aeruginosa* compared to the individual use of each agent [14]. In contrast, the combined use of LLKKK-18 and AgNPs demonstrated a reduced bactericidal effect against *Mycobacterium smegmatis*, suggesting that their actions might interfere with each other, resulting in antagonistic effects [15]. In this study, we examined the combined effects of P1 and AgNPs on *S. aureus* and *E. coli* through bacterial growth inhibition assay, time–kill study, and morphological changes in bacterial cells. Additionally, cytotoxicity and in vitro wound healing assays were conducted to evaluate the safety profile and potential use of the tested combinations.

## 2. Results and Discussion

### 2.1. Preparation and Characterization of AgNPs

The AgNPs were synthesized through a redox reaction between AgNO_3_ and trisodium citrate. The citrate was a reducing and stabilizing agent that reduced AgNO_3_ to AgNPs. The chemical reaction is presented as follows (1):

(1) $$4{\mathrm{Ag}}^{+}+\;C_{6}H_{5}O_{7}{\mathrm{Na}}_{3}+\;2H_{2}O\;\to \;4\mathrm{Ag}\;+\;C_{6}H_{5}O_{7}H_{3}+\;3{\mathrm{Na}}^{+}+\;H^{+}+O_{2}$$

**Figure 1:**
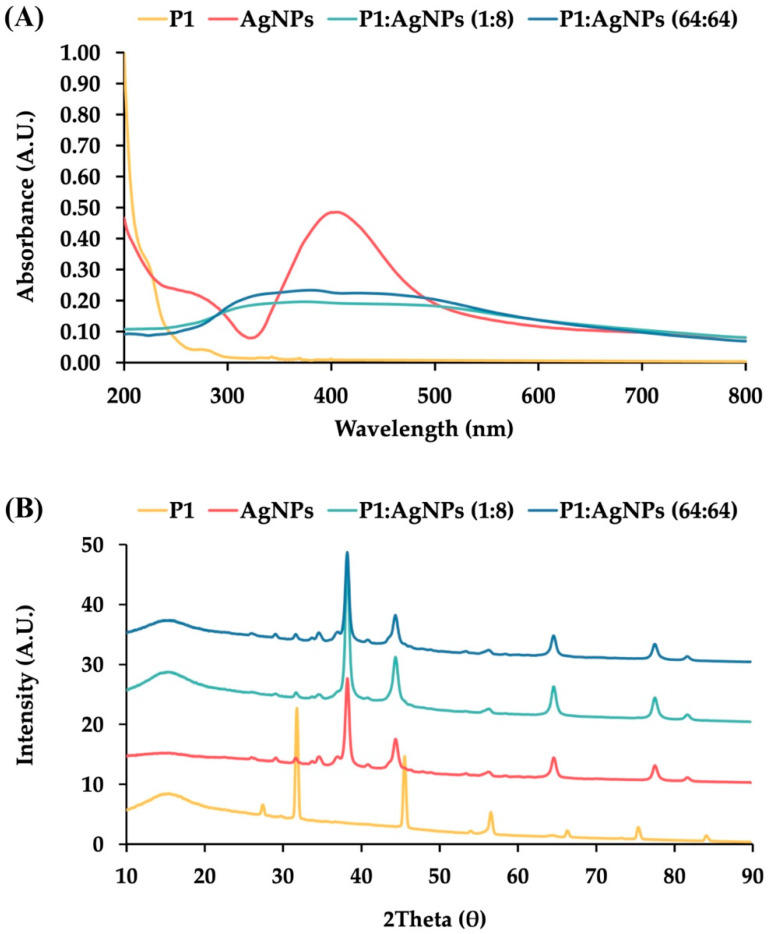
Characterization of P1, AgNPs, and their combinations. (**A**) UV—visible (UV—vis) absorption spectra and (**B**) Powder X-ray diffraction patterns of P1 (yellow line), AgNPs (red line), and their mixtures at concentrations of 1:8 μg/mL (light blue line) and 64:64 μg/mL (dark blue line).

AgNPs had a light yellow color in a transparent solution (Figure S1). The color change in metallic nanoparticles is typically caused by the interaction of electromagnetic radiation and electrons at the particle surface, resulting in a shift in the absorbance spectrum to a higher wavelength, confirming the formation of AgNPs [16]. Our result showed that AgNPs had an absorbance peak at 414.0 ± 1.2 nm (Figure 1A). It was consistent with other studies, demonstrating the absorbance maxima of AgNPs between 400 and 500 nm [17]. Moreover, the spectral shift was dependent on particle size and shape, affecting the color of colloidal liquid from yellow to brown [18]. Generally, larger nanoparticles with spherical shapes produced a peak at a higher wavelength. The Mie scattering theory was used to calculate the particle size based on the absorbance peak, and it was found that the synthesized AgNPs had a size of 37.7 ± 1.1 nm [19].

Transmission electron microscopy (TEM) revealed that AgNPs were spherical with an average diameter of 26.7 ± 11.5 nm (Figure S2). It corresponded with the previous results, showing that the spherical AgNPs had a size between 15 and 50 nm [20]. The results from Zetasizer demonstrated that AgNPs had an average hydrodynamic size of 46.3 ± 0.5 nm with a polydispersity index (PDI) of 0.578 ± 0.004 (Table 1). The PDI value is an indicator of the heterogeneity of particle size in the sample preparations. It scales from 0 to 1 by which a lower value indicates a monodisperse system, whereas a higher value represents polydispersity. Our PDI was higher than 0.5, indicating polydispersity, which referred to a broad size distribution of AgNPs. It supported the result of the TEM analysis, showing a size range of AgNPs from 3 to 57 nm. The particle size of AgNPs measured by Zetasizer was larger than that measured by TEM, possibly because of the solvation effect in the sample solution, which resulted in a larger hydrodynamic diameter compared to other methods [21]. Moreover, this study found that AgNPs had a zeta potential of −20.58 ± 0.69 mV. Zeta potential is a parameter for estimating the electrokinetic potential at the shear plane of the particles in the dispersion solvent. Generally, a high zeta potential value (greater than ±30 mV) represents high stability of colloidal dispersion influenced by strong electrostatic repulsions of the same charged particles. Nanoparticles with low zeta values (between −10 and +10 mV) are highly unstable and promote rapid agglomeration. The moderate stability of nanoparticles occurs at zeta potential between ±20 to ±30 mV [22]. The negative zeta potential in this study was caused by the accumulation of negatively charged citrate surrounding the surface of silver nanoparticle clusters. The negative charges due to this stabilizing agent also contributed to AgNP stability by preventing particle aggregations [20]. This result was similar to a previous study using citrate as a stabilizer in the synthesis of AgNPs, yielding a small particle size (16 ± 5 nm) and negative zeta potential (−25.6 mV) [23].

The powder X-ray diffraction (PXRD) of AgNPs showed five diffraction peaks at 2θ of 38.18°, 44.42°, 64.63°, 77.58°, and 82.02°, corresponding to the Miller indices of (1 1 1), (2 0 0), (2 2 0), (3 1 1), and (2 2 2), respectively (Figure 1B). These reflection planes corresponded to the crystalline silver with a face-centered cubic structure (JCPDS file no. 04-0783) [24]. The average crystalline size was calculated by the Scherrer equation. It was found that AgNPs had a coherent scattering region of 15.0 ± 1.3 nm, suggesting that they were polycrystalline nanoparticles, composed of multiple crystals rather than a single large crystal [25].

### 2.2. Antimicrobial Activity of P1 and AgNPs

P1 was an AMP produced from *Brevibacillus* sp. SPR20. It showed strong activity against Gram-positive bacteria (*S*. *aureus* TISTR 517 and MRSA isolate 246) with minimum inhibitory concentration (MIC) and minimum bactericidal concentration (MBC) at 2 and 4 µg/mL, respectively (Table 2). P1 was comparable to vancomycin, exhibiting similar antibacterial activity. Moreover, the high concentration of P1 (256 µg/mL) was observed to inhibit and kill *E*. *coli* TISTR 887, suggesting that it was less active against Gram-negative bacteria. When ampicillin was used as a positive control for *E*. *coli* TISTR 887, the MIC and MBC were 4 and 8 µg/mL, respectively. AgNPs had MIC and MBC values of 256 µg/mL against all tested bacteria. Our previous study revealed that P1 had antibacterial efficacy through membranolytic activity [9]. Therefore, it might suggest that the difference in cell wall structure and membrane composition contributed to the various potencies of AMPs. Gram-negative bacteria have an additional outer membrane as a barrier, preventing AMPs from reaching the inner membrane. Other factors, such as the modification of lipopolysaccharides, the activation of efflux pumps, and the presence of proteases, could also cause different killing effects of AMPs [26]. This might explain why a high concentration of P1 was required to eliminate Gram-negative bacteria. Similarly, daptomycin was a membrane-active peptide that disrupted the cytoplasmic membrane but could not interrupt the outer membrane. As a result, it was more potent against Gram-positive bacteria such as *S*. *aureus* and MRSA but showed no activity against Gram-negative bacteria such as *E*. *coli* [27]. Moreover, AgNPs have been demonstrated to be a broad-spectrum bactericidal agent. Their particle size plays a crucial role in antimicrobial activity, in which the linear relationship between antimicrobial activity and nanoparticle size was observed [28]. The smaller the particle size, the higher the antimicrobial activity. For example, the citrate-functionalized AgNPs with particle sizes of 7, 10, 20, and 50 nm exhibited the MIC against *E*. *coli* at 0.18, 0.28, 0.37, and 0.56 mM, respectively [28]. Similarly, nanoparticles with a small size (38.5 nm) released more Ag^+^ than larger particles (51.1 nm), showing superior antimicrobial activity [29]. Several explanations were provided to support this finding. Small nanoparticles can easily penetrate bacterial cells and have a large surface area, facilitating the high release rate of Ag^+^ into the solution. Furthermore, Ag^+^ can inhibit respiratory enzymes and increase intracellular concentrations of reactive oxygen species (ROS), resulting in bacterial cell damage. A recent investigation revealed that a combined ion-particle action was the mechanism of small AgNPs (10 nm) on bactericidal activity, using synergistic effects between the released silver ions and the intracellular penetrations of nanoparticles. The release of ionic silver was only responsible for the action of large AgNPs (60 nm) without cellular uptake of particles [30].

### 2.3. Synergistic Effect of P1 and AgNP Combination

**Figure 2:**
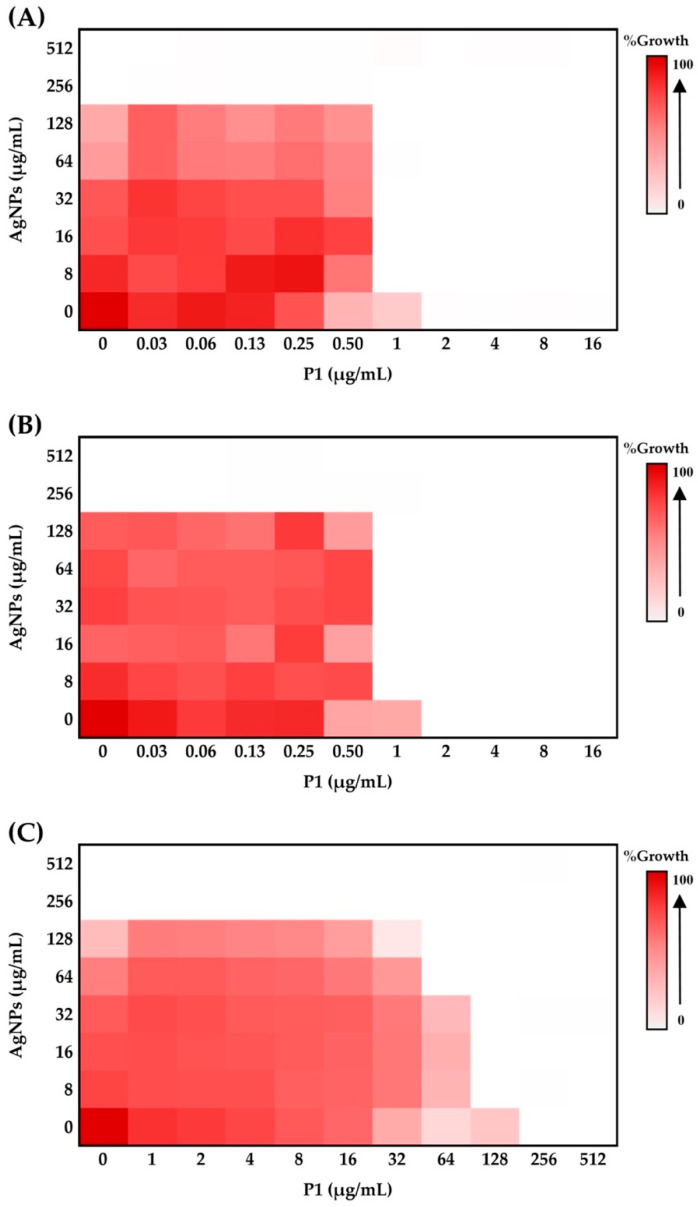
Effect of P1 and AgNPs at different concentrations on bacterial growth. (**A**) *S*. *aureus* TISTR 517, (**B**) MRSA isolate 2468, and (**C**) *E*. *coli* TISTR 887. Various P1 and AgNP combinations (0–512 µg/mL) affected the bacterial growth. A heatmap was utilized to visualize cell growth across different combinations. White indicates no growth, while red represents a high growth level. The concentrations of P1 and AgNPs in the figure represent the final concentrations of each substance included in the mixture.

Many combinations of antimicrobial agents interestingly provide synergy, exhibiting greater antimicrobial efficacy, reducing drug toxicity, and preventing the emergence of drug resistance. A checkerboard assay was used to evaluate the impact on antibacterial activity of P1 and AgNP combinations by determining the fractional inhibitory concentration index (FICI). The combination with the lowest FICI value will be selected for analysis. It was revealed that bacterial inhibition was dependent on the concentration of either P1 or AgNPs. High concentrations of P1 or AgNPs resulted in no growth of the tested bacteria (Figure 2). Bacterial growth declined when P1 (0.03–512 µg/mL) and AgNPs (8–512 µg/mL) were combined. The P1 and AgNP mixture at a final concentration of 1 and 8 µg/mL, respectively (referred to as the 1:8 mixture), inhibited *S*. *aureus* TISTR 517 and MRSA isolate 2468 and gave the minimal FICI of 0.5, indicating a synergistic effect (Figure 2A,B). The mixture decreased the MIC value compared to the effect of individual components, showing higher antibacterial activity with a 2-fold change based on P1 (Table 2). However, the P1 and AgNP combination at a 1:8 µg/mL concentration failed to inhibit *E. coli* TISTR 887. It was only synergistically effective at high concentrations of P1 and AgNPs, specifically at 64 and 64 µg/mL (referred to as the 64:64 mixture) and at 128 and 8 µg/mL, with a minimal FICI of 0.5, indicating a 32- and 2-fold reduction in MIC, respectively (Figure 2C). Therefore, P1 exhibited strong efficacy against the tested Gram-positive bacteria. Its effectiveness increased and extended to the tested Gram-negative bacteria when combined with AgNPs. This synergistic effect might occur through complementary mechanisms by which each substance functions on different targets. As mentioned earlier, AgNPs exert antibacterial activity via multiple mechanisms, including the disruption of cell walls, the release of Ag^+^, and the generation of ROS. AMPs typically target the inner membranes of bacteria, exhibiting excellent activity towards Gram-positive bacteria. These peptides showed less activity against Gram-negative bacteria because the outer membranes, comprising lipopolysaccharide (LPS), a barrier layer, limited their penetration into the inner membrane. Consequently, AgNPs could enhance the P1 activity by altering the cell walls and outer membranes and enhancing AMP action on the inner membrane. These mutual interactions might also enhance the cellular penetration of P1 and AgNPs, leading to bacterial killing through their intracellular activity [31].

Several investigations have demonstrated the synergistic interaction between AMPs and AgNPs. For example, polymyxin B and AgNPs exhibited the greatest synergy for Gram-negative bacteria, with FICI values ranging from 0.23 to 0.39. This synergy is attributed to the ability of polymyxin B to permeabilize the outer bacterial membrane, thereby enhancing the intrinsic antibacterial activity of AgNPs [13]. The antibacterial activity of colistin (10 µg) with AgNPs (100 µg) was also observed against Gram-negative bacteria using the disk diffusion assay. The diameter of the inhibition zone produced by the combined mixture was greater than that of colistin alone, suggesting a synergistic effect between colistin and AgNPs. It was postulated that colistin interrupted the bacterial outer membrane, allowing AgNPs to penetrate the cells. The production of ROS by nanoparticles altered bacterial DNA, proteins, and lipids, ultimately resulting in cell death [32]. Moreover, the synergy between kanamycin (0.05–16 µg/mL) and AgNPs (6 µg/mL) was confirmed by FICI values ranging from 0.35 to 0.47. The mechanism underlying this combination involves AgNP-induced depolarization of bacterial membranes and the disruption of cellular integrity in the tested pathogens, including *S. aureus*, *E. coli*, and *Salmonella Typhimurium*. This allowed the permeability of the drug to target the ribosome and enhanced antibacterial activity [33]. In this study, the P1 and AgNP combinations at concentrations of 1:8 and 64:64 µg/mL, which exhibited the greatest reduction in MIC, were selected to evaluate their antibacterial activity against the tested Gram-positive and Gram-negative bacteria, their potential for inducing antibacterial resistance, and their safety.

### 2.4. Physical Characterization of P1 and AgNP Mixture

To characterize the physical interactions between P1 and AgNPs identified from the checkerboard assay, UV–vis spectra of the 1:8 and 64:64 mixtures exhibited broadened absorption peaks and decreased intensity (Figure 1A). These spectral changes in the P1 and AgNP mixtures were attributed to surface interference on the nanoparticles, where peptides displaced citrate molecules on the AgNP surface. This explanation was supported by a previous study, which demonstrated that certain pentapeptides, such as CLKRS, CLFRS, and SLKRS, significantly altered the absorbance spectra of AgNPs by reducing the absorbance intensity and shifting the peak wavelength from 400 to 430 nm. [34]. The study demonstrated that these pentapeptides replaced citrate on the nanoparticle surface, resulting in broadened UV–vis spectra due to loss of the stabilizing agent, aggregation of unstable AgNPs, and the formation of a peptide corona around the nanoparticle surface.

**Figure 3:**
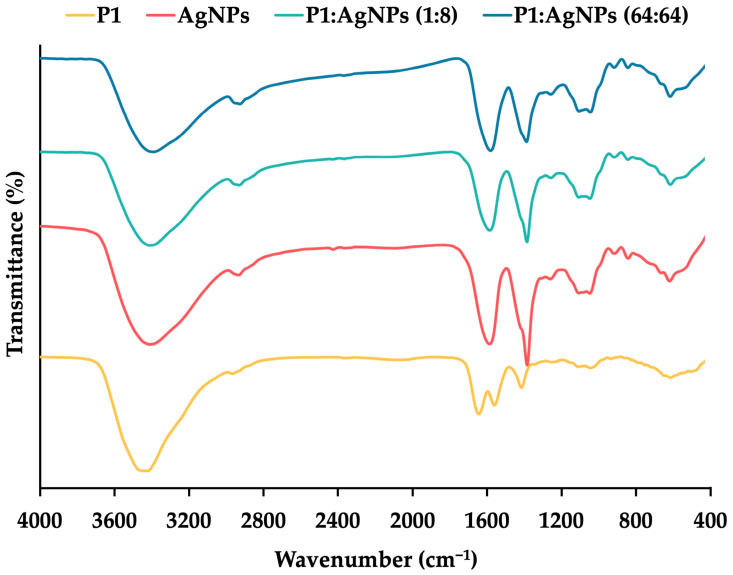
FT–IR spectra of P1 (yellow line), AgNPs (red line), and their mixtures at concentrations of 1:8 μg/mL (light blue line) and 64:64 μg/mL (dark blue line).

Fourier transform-infrared spectroscopy (FT–IR) was used to study the interaction between P1 and AgNPs in the mixtures (Figure 3). The result found the presence of O–H stretching at 3424 cm^−1^, C–H stretching at 2966 cm^−1^, C=O stretching at 1644 cm^−1^, and N–H bending at 1558 cm^−1^ for P1. The spectrum of AgNPs exhibited stretching vibrations of O–H, C–H, and C=O at 3409, 2935, and 1603 cm^−1^, respectively. The presence of citrate as a stabilizing agent in the AgNPs contributed to these characteristic wavenumbers. It was consistent with a previous study, showing a similar FT–IR spectrum because of the binding of citrate on AgNPs [35]. Both the 1:8 and 64:64 combinations exhibited corresponding stretching vibrations in the ranges of 3391–3409 (O–H), 2927–2931 (C–H), and 1607–1609 (C=O) cm^−1^, respectively. The N–H bending (amide II) was shifted in the spectra of the mixtures, suggesting an interaction between P1 and AgNPs [36].

The PXRD result showed that P1 exhibited distinct diffraction peaks, indicating that the peptide molecules formed a crystalline structure (Figure 1B). The mixtures of P1 and AgNPs at concentrations of 1:8 and 64:64 μg/mL displayed prominent peaks corresponding to crystalline silver. It indicated that P1 did not change the crystal structure of silver. However, the characteristic peaks of P1 disappeared in the mixtures, possibly resulting from a small amount of P1 or loss of peptide crystallinity caused by surface interactions upon binding to AgNPs. Previous studies have reported different XRD patterns for mixtures of peptides and AgNPs, such as the presence of dominant crystalline silver peaks after coating AgNPs with casein hydrolysate peptides, and a reduction in the intensity of AgNP peaks following conjugation with MT-6 and CUTP-1 peptides [37,38].

Zeta potential analysis was performed to evaluate changes in the surface charge of the nanoparticles. P1 exhibited a slightly positive zeta potential of 0.23 ± 0.22 mV. When combined with AgNPs, the zeta potentials of the 1:8 and 64:64 mixtures decreased to −11.39 ± 0.57 and −7.49 ± 0.52 mV, respectively, which can be attributed to the positively charged nature of P1 influencing the overall surface charge (Table 1). Additionally, P1 displayed a hydrodynamic diameter of 439.4 ± 34.0 nm. Upon mixing with AgNPs, the particle size increased substantially, ranging from 2275.3 ± 0.6 to 2700.7 ± 1.1 nm, along with an increase in PDI from 0.667 ± 0.096 to 0.709 ± 0.050. These findings suggest that electrostatic interactions between P1 and AgNPs led to partial charge neutralization, promoted nanoparticle aggregation, and increased size heterogeneity. Our results are consistent with previous studies, which reported that peptide-coated silver nanoparticles (MFP-AgNPs) exhibited lower zeta potential compared to unmodified AgNPs, confirming successful surface functionalization with peptides [39]. The increased particle size of the P1 and AgNP mixtures, as determined by Zetasizer analysis, was consistent with the UV-vis and FT–IR results, further supporting the occurrence of physical interactions between P1 and AgNPs in the mixtures.

### 2.5. Bacterial Killing Kinetics

**Figure 4:**
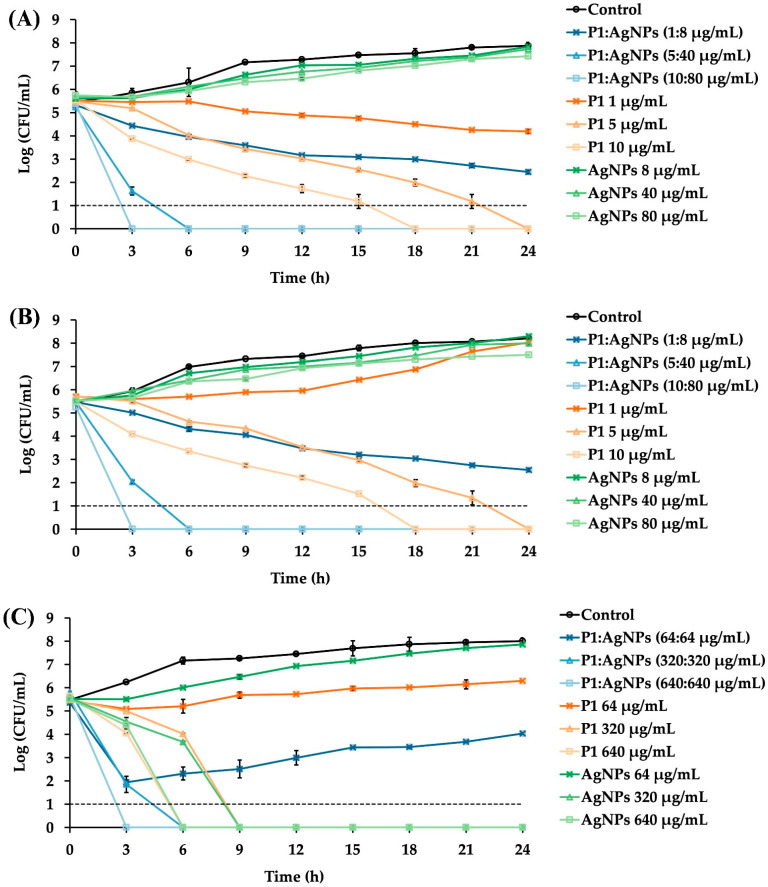
Time–kill kinetics of P1, AgNPs, and their combinations. Different P1 and AgNP combinations at 1×, 5×, and 10× MIC were incubated with the tested bacteria. (**A**) *S*. *aureus* TISTR 517 and (**B**) MRSA isolate 2468 were cultured in the presence of the P1 and AgNP combination at a 1:8 µg/mL concentration. (**C**) *E*. *coli* TISTR 887 was incubated with P1 and AgNPs at 64:64 µg/mL. The growth of untreated bacteria and treated bacteria with either P1 or AgNPs was also assessed. Each sample at the specified time points underwent serial dilution and plate counting. The limit of detection of the assay was 10 CFU/mL and is represented as the dashed line.

The P1 and AgNP combinations significantly reduced bacterial growth over the study period compared to the untreated samples. These combinations at 1× MIC failed to eradicate the tested bacteria. The inhibition of Gram-positive bacteria by the 1:8 mixture of P1 and AgNPs gradually declined over 24 h (Figure 4A,B). In contrast, Gram-negative bacteria treated with the 64:64 mixture of P1 and AgNPs showed a sharp reduction within the first 3 h but tended to grow again over time (Figure 4C). *E. coli* typically grows faster than *S. aureus*. Therefore, the growth of *E. coli* was inhibited at 1× MIC, but this concentration was insufficient to kill the bacteria completely. Some bacteria survived, allowing for a subsequent cell recovery [40]. These findings also indicated that all tested combinations at 1× MIC exhibited a bacteriostatic effect rather than bactericidal activity. Consequently, the cells could resume their growth when the antimicrobial concentration decreased. Compared to the P1 and AgNP combination at 1× MIC, treatment with either P1 or AgNPs alone at an equivalent concentration did not result in the suppression of bacterial growth. Other studies observed a rapid recovery of bacteria after 6–8 h of treatment. For instance, the time–kill curve of *Klebsiella pneumoniae*, incubated with a polymyxin B derivative and antibiotics (clarithromycin and erythromycin), showed bacterial growth again. This might have been caused by the decreased peptides and antibiotics, the presence of a resistant bacterial subpopulation, and inoculum effects [41]. Additionally, AgNPs were found to prolong the log phase of bacterial growth. As a result, bacterial proliferation was delayed during the initial period, and the cells continued to undergo normal binary fission, resulting in bacterial growth when the antimicrobial concentration decreased over time [42]. In contrast, the P1 and AgNP combinations at 5× MIC showed bacterial elimination within 6 h for all tested Gram-positive and Gram-negative bacteria. However, treatment with either P1 or AgNPs alone at an equivalent amount of the combinations at 5× MIC showed different inhibition. P1 (5 or 320 μg/mL) or AgNPs (320 μg/mL) killed all bacteria because their concentrations exceeded MBC, whereas AgNPs (40 μg/mL) could not inhibit Gram-positive bacteria. At 10× MIC values, all combinations successfully eradicated the tested bacteria within 3 h. Moreover, individual treatment with either P1 or AgNPs at the corresponding concentration killed all bacteria, except AgNPs at 80 μg/mL, which did not suppress bacterial growth because its concentration was lower than MBC.

The killing rate of the P1 and AgNP mixtures within the first 3 h was calculated to compare the activity of antimicrobial combinations. The killing rates for *S. aureus* TISTR 517 were 0.69 ± 0.06, 2.78 ± 0.14, and 4.13 ± 0.04 h^−1^, while those of the MRSA isolate 2468 were 0.35 ± 0.01, 2.63 ± 0.05, and 4.04 ± 0.01 h^−1^ at 1×, 5×, and 10× MIC of the 1:8 mixtures, respectively. Interestingly, the killing rates of MRSA were lower than those of *S*. *aureus*, implying that the resistant characteristics involved a slow bactericidal effect when treated with these mixtures. For *E*. *coli* TISTR 887, the killing rate of the 64:64 mixture was 2.62 ± 0.13 h^−1^ at 1× MIC, 3.03 ± 0.28 h^−1^ at 5× MIC, and 4.31 ± 0.03 h^−1^ at 10× MIC. These results indicated that the antibacterial efficacy of the P1 and AgNP combinations was concentration-dependent, and a higher killing rate occurred at higher concentrations of the combination. The mixture at concentrations above 1× MIC could enable more than three-log reductions in cell growth, relative to the initial inoculum, supporting the bactericidal activity of such combinations at higher concentrations [43]. A comparable bactericidal effect was observed when bacterial cells were treated with either P1 or AgNPs at concentrations above their 1× MIC, although the killing rate was slower than that observed with their combination. Moreover, when compared to standard antibiotics, a previous study showed that vancomycin exhibited bactericidal activity against *S. aureus*, but its activity was concentration-independent. The killing was observed after 4 h and continued up to 24 h of incubation to cause more than three-log reductions, implying a slow killing rate of vancomycin (about 0.38 h^−1^) [44]. Moreover, ampicillin was a bactericidal agent for *E. Coli*, by which the bacterial growth reached zero after a 1 h exposure to the antibiotic. The onset of the bactericidal effect was concentration-dependent, and the killing rate of ampicillin at 256 µg/mL was 4.0 ± 0.8 h^−1^ [45]. The higher the killing rate, the faster the bacterial eradication. Thus, the synergy of P1 and AgNPs at high concentrations provided rapid bacterial killing within 3 h and might offer several benefits, such as reduced risk of resistance, shorter treatment duration, and rapid infection control [46].

### 2.6. Development of Antimicrobial Resistance

**Figure 5:**
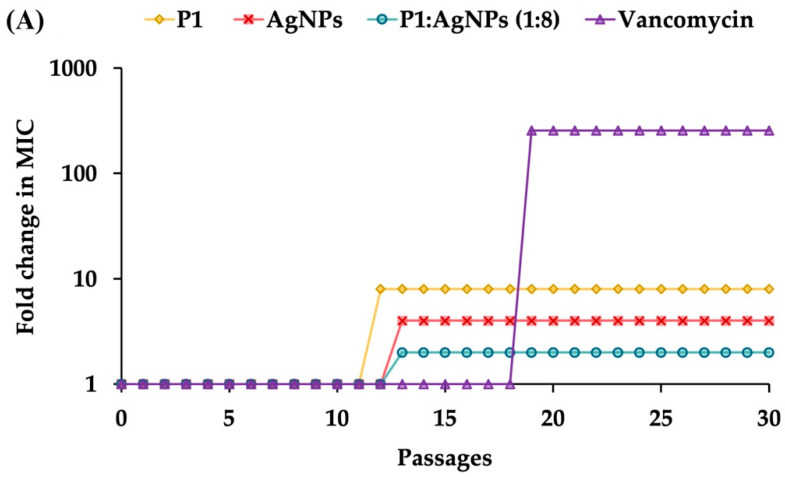

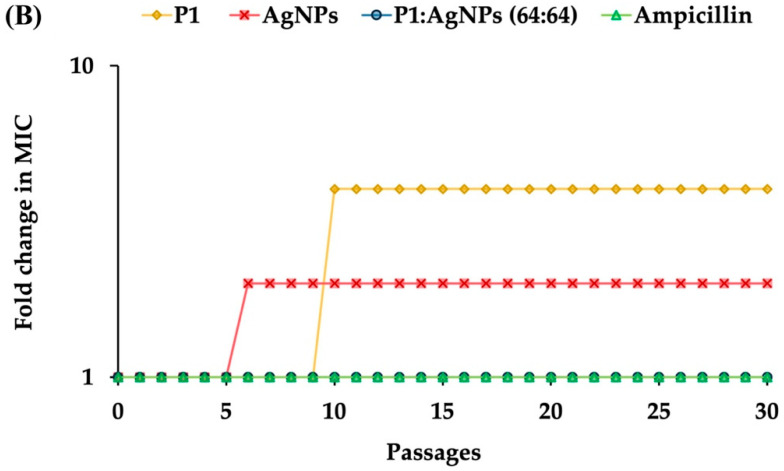
Effects of the P1, AgNPs, and their combinations on the induction of bacterial resistance. The samples at sub-MIC were incubated with (**A**) *S*. *aureus* TISTR 517 and (**B**) *E*. *coli* TISTR 887. The cells were subcultured for 30 passages, and their MIC values were assessed.

To evaluate the potential of the P1 and AgNP combinations to induce antimicrobial resistance, *S. aureus* TISTR 517 and *E*. *coli* TISTR 887 underwent sub-MIC exposure (0.5× MIC), followed by sequential sub-culturing and repeated exposure to the same dose for 30 passages. The results demonstrated that sustained exposure to the 1:8 combination showed a low propensity to induce resistance in *S. aureus* TISTR 517 (Figure 5A). Specifically, this combination at 2× MIC was required to inhibit bacterial growth after passage 13. In comparison, vancomycin developed high resistance levels since passage 19, which used 256× MIC to suppress bacterial proliferation. The slow killing rate of vancomycin may be one of the explanations for this situation, by which the drug at sublethal concentrations prolongs bacterial exposure, potentially promoting the development of resistance mechanisms [44]. Moreover, cell exposure with P1 or AgNPs alone resulted in a moderate resistance in *S*. *aureus* TISTR 517, as indicated by the requirement of 8× MIC of P1 or 4× MIC of AgNPs to inhibit bacterial growth after passage 12 or 13, respectively. Additionally, the underlying resistance mechanisms in *S. aureus* may involve multiple pathways, including peptidoglycan modification, production of antibiotic-inactivating enzymes, and efflux pump activation. A study evaluated the resistance of *S. aureus* treated with an antimicrobial peptide AP138, showing that MIC was elevated 8-fold. The resistant genes, such as *dltA* and *mprF*, were identified in this resistant strain [47]. The *dltA* gene modifies teichoic acids in the bacterial cell walls, reducing the negative charges on the surface of *S. aureus*. Meanwhile, the *mprF* gene alters the composition of the bacterial membrane. These changes decrease the binding of cationic AMPs to the cell surface and confer resistance to AMPs. Moreover, the 64:64 mixture and ampicillin did not induce antimicrobial resistance in *E. coli* TISTR 887 throughout the study period (Figure 5B). The faster killing of these compounds resulted in a rapid bactericidal effect. This helps reduce the emergence of antimicrobial-resistant bacteria by minimizing their exposure to subinhibitory concentrations of antimicrobials. This also prevents adaptive responses, like biofilm formation and efflux pump activation, which bacteria use to survive treatment. In contrast, individual administration of P1 or AgNPs resulted in the development of resistance in *E. coli* TISTR 887, as evidenced by the requirement of 4× MIC of P1 or 2× MIC of AgNPs to suppress bacterial growth after passage 10 or 6, respectively. Therefore, the simultaneous use of P1 and AgNPs enhanced their antibacterial efficacy and caused low or no resistance through rapid actions on different bacterial targets.

### 2.7. Effect of the Combinations on the Morphology of the Treated Bacteria

**Figure 6:**
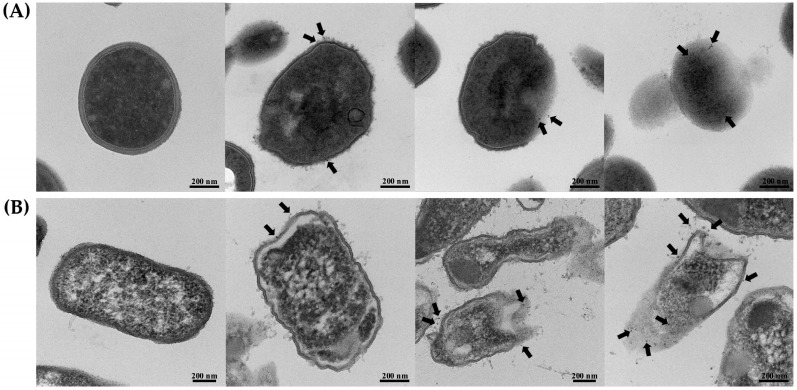
TEM images of the treated bacteria. (**A**) *S. aureus* TISTR 517 and (**B**) *E. coli* TISTR 887 were incubated with the P1 and AgNP combinations at the concentration of 1:8 and 64:64 µg/mL, respectively. The micrographs from different stages were taken at 36,000× or 46,000× magnification. Black arrows indicate the presence of AgNPs.

**Figure 7:**
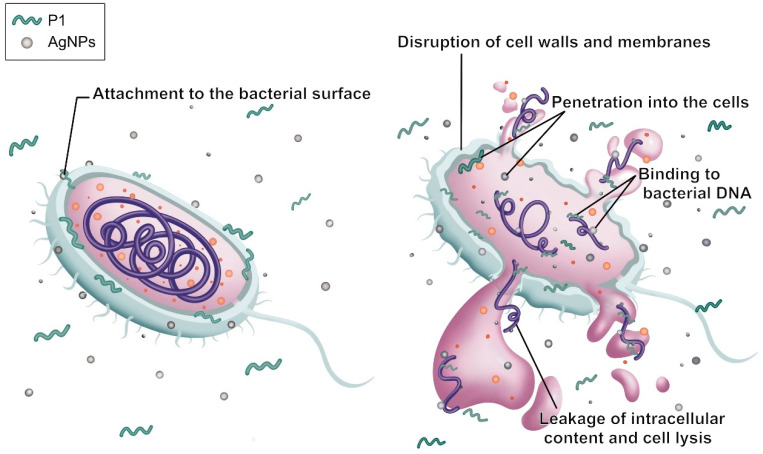
Proposed synergistic mechanism of P1 and AgNPs on the bacterial cell. The gray sphere corresponds to AgNPs, the green folded line depicts the P1 peptide, and the orange sphere together with the purple line represent bacterial cellular components.

The morphological characteristics of *S*. *aureus* TISTR 517 and *E. coli* TISTR 887 upon exposure to the P1 and AgNP combinations were examined by TEM. The results revealed that both cells had intact cell structures in the untreated condition. When the cells were incubated with the combinations, the AgNPs (arrows) were deposited at the bacterial membrane (Figure 6A,B). Subsequently, the cell walls were ruptured, and the intracellular components leaked. Generally, Gram-positive bacteria have simple cytoplasmic membranes and a thick and porous peptidoglycan layer that contains teichoic and lipoteichoic acids. Gram-negative bacteria have a thin peptidoglycan with an outer layer of LPS, making them more resistant to antimicrobial agents. Therefore, *S. aureus* TISTR 517 was more sensitive to the P1 and AgNP combination than *E. coli* TISTR 887 due to differences in their cell wall structures and compositions. Based on the TEM of the treated cells, it is postulated that AgNPs attach to the cell walls and alter cell permeability, facilitating the penetration of P1 to the inner membrane. In addition, the entry of P1 and AgNPs into the bacterial cells enables intracellular activities, leading to cell death (Figure 7). However, the exact mechanisms of the combination of P1 and AgNPs need further investigation. Our findings were consistent with several reports that bacteria exposed to AgNPs exhibited irregular cell morphology, membrane disruption, and DNA damage, leading to cellular demise [48]. Furthermore, the combination of bacteriocin and AgNPs showed synergistic antibacterial efficacy against multidrug-resistant bacteria. The mechanisms of this bactericidal activity involved the alteration of membrane permeability, leakage of proteins and DNA, and ROS production [49].

### 2.8. Effect of the P1 and AgNP Mixtures on Bacterial Genomic DNA

**Figure 8:**
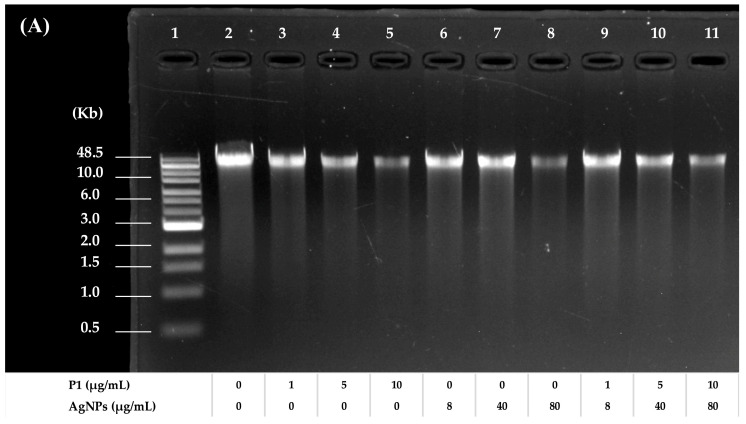

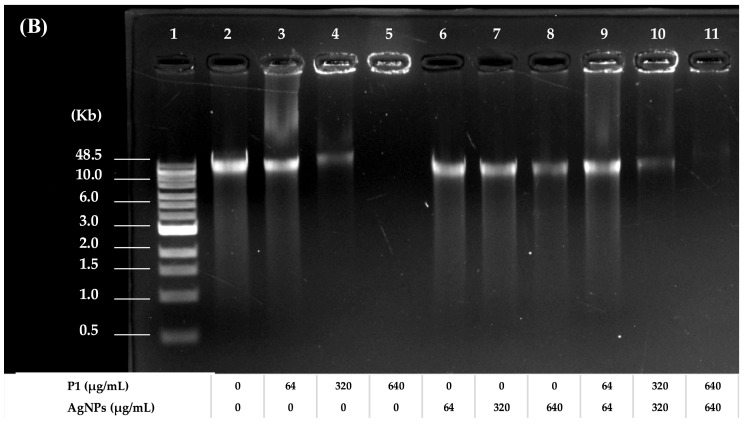
Agarose gel electrophoresis of treated and untreated bacterial genomic DNA. The electrophoretic mobility of DNA from (**A**) *S. aureus* TISTR 517 and (**B**) *E. coli* TISTR 887 after treatment with P1 and AgNPs at the 1:8 and 64:64 combinations, respectively. Lane 1: DNA marker. Lane 2: untreated DNA. Lanes 3–5: DNA treated with different concentrations of P1. Lanes 6–8: DNA treated with different concentrations of AgNPs. Lanes 9–11: DNA treated with different concentrations of the P1 and AgNP combinations.

To evaluate the effects of P1, AgNPs, and their combinations on DNA, an electrophoretic mobility shift assay was employed using bacterial genomic DNA as a mobility shift indicator. The results revealed that the band intensity of DNA treated with P1 or AgNPs decreased when DNA was incubated with an increasing concentration of individual substances. The bands were retarded in an agarose gel, and some DNA was deposited in the wells. The remaining intensity of the genomic DNA decreased from 86.52 ± 6.15% to 44.73 ± 5.24% (Figure 8A) and from 69.87 ± 9.65% to 8.39 ± 0.82% (Figure 8B) when incubated with P1 at concentrations ranging from 1 to 10 μg/mL and 64 to 640 μg/mL, respectively. Moreover, AgNPs at concentrations ranging from 8 to 80 μg/mL and 64 to 640 μg/mL reduced DNA intensity from 91.22 ± 4.26% to 44.37 ± 5.39% and 71.73 ± 2.46% to 42.69 ± 9.26%, respectively. The interactions between P1 or AgNPs and DNA produced higher molecular weight complexes. This resulted in a slow migration of treated DNA compared to the untreated DNA. Similarly, the P1 and AgNP combinations (1:8 and 64:64) caused an obvious band shift. Especially when DNA was combined with high concentrations of the mixtures at 64:64 µg/mL, the DNA intensity was significantly reduced by 43.26 ± 6.13%, 81.68 ± 2.24%, and 95.80 ± 3.43% when incubated with those mixtures at 1×, 5×, and 10× MIC, respectively. Similarly, treatment with the 1:8 mixtures resulted in reductions in DNA intensity of 25.84 ± 7.27%, 41.71 ± 8.10%, and 63.14 ± 0.14% at 1×, 5×, and 10× MIC, respectively. Therefore, the P1 and AgNP combinations at high concentrations might contribute to greater interaction with bacterial genomic DNA. These findings corresponded with previous studies, demonstrating that AgNPs interacted with DNA, interfering with replication and transcription and ultimately leading to cell death [50]. AgNPs also induced ROS, leading to DNA strand breaks and fragmentations. Thus, DNA damage is a key factor contributing to antimicrobial activity. Moreover, certain AMPs inhibited or killed bacteria by binding to intracellular nucleic acids, disrupting essential cellular functions necessary for genetic integrity and bacterial survival. For instance, the cationic A11 peptide at a concentration of more than 1× MIC (15.63 μg/mL) remarkably retarded the migration of genomic DNA of *Acinetobacter baumannii*, possibly contributing as either a primary or supportive mechanism to exert their bactericidal activity [51]. Consequently, the synergistic actions of P1 and AgNPs might support their bactericidal potential through disruption of genomic integrity.

### 2.9. Hemolysis Activity

**Figure 9:**
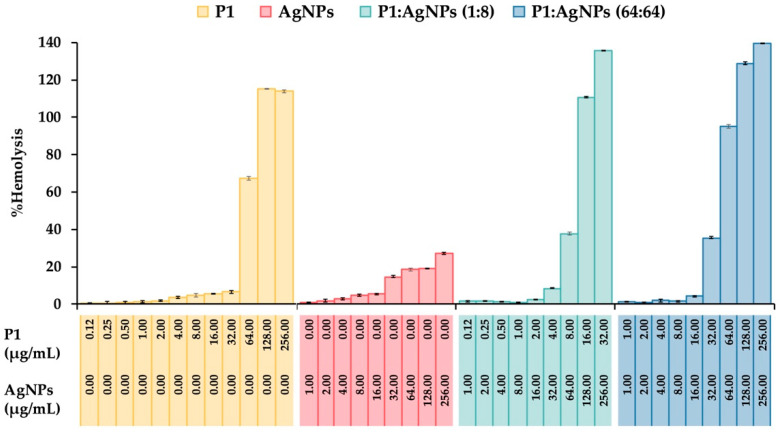
Hemolytic activity of the P1, AgNPs, and their combinations. The effect of different concentrations of P1, AgNPs, and their combinations at 1:8 and 64:64 µg/mL on the lysis of human erythrocytes was evaluated. Triton X-100 (1%) and phosphate-buffered saline (PBS) were used as positive and negative controls, respectively. The concentrations of P1 and AgNPs in the figure represent the final concentrations of each substance included in the mixture.

A hemolysis assay was conducted to evaluate the toxicity of substances by measuring hemoglobin release after exposing human erythrocytes to P1, AgNPs, and their combinations. The results showed that the degree of hemolysis increased when the erythrocytes were treated with higher concentrations of such substances. Generally, the substances that cause a hemolysis of less than 10% are considered safe for therapeutic applications. P1 alone at concentrations between 0.12 and 32 µg/mL showed hemolytic activity, ranging from 0.47 ± 0.18% to 6.49 ± 0.70% (Figure 9). AgNPs alone (1–16 µg/mL) demonstrated the percent hemolysis between 1.00 ± 0.18% and 5.38 ± 0.45%. The selective index (SI) for hemocompatibility was calculated based on the highest concentration of the substance that caused not more than 10% hemolysis and its respective MIC. It was found that P1 and AgNPs had the SI of 16 and 0.062, respectively, indicating that AgNPs were more toxic to erythrocytes than P1. Moreover, the P1 and AgNPs combination (1:8) gave the hemolytic activity of 8.39 ± 0.47% at the concentration of 4:32 µg/mL, providing the SI of 4. The hemocompatibility of the mixture (64:64) was observed at the highest concentration of 16:16 µg/mL with an activity of 4.20 ± 0.31%, having the SI of 0.25. These findings suggested that the toxicity of P1 and AgNP combinations was dose-dependent. Lower concentrations of the P1 and AgNP mixtures were hemocompatible, whereas higher concentrations may require dose adjustments to minimize hemolytic effects. Several investigations have explained the mechanisms of AMPs and AgNPs on hemolysis. It demonstrated that hydrophobicity and charge affect the selectivity of AMPs. AMPs with high positive charge and hydrophobicity could interact and insert into the phospholipid membrane of erythrocytes, leading to membrane destabilization and hemolysis. Magainin-2 analogs with the extension of 4–20 positively charged lysine to their amino or carboxyl terminus were found to enhance the antimicrobial activity and hemolysis than their original peptide [52]. The addition of 1–5 hydrophobic tryptophans at the termini of GKH17 enhanced the bactericidal potency and hemolytic effect [53]. Furthermore, AgNPs induced hemolysis in a size- and dose-dependent manner. The small particles with 15 nm caused higher hemolysis than the large ones, and a higher number of particles induced more hemoglobin release [54]. It is assumed that the mechanism by which AgNPs cause hemolysis involves direct interaction between particles and the cell membrane of erythrocytes, along with the release of free silver ions.

### 2.10. Cytotoxicity Test

**Figure 10:**
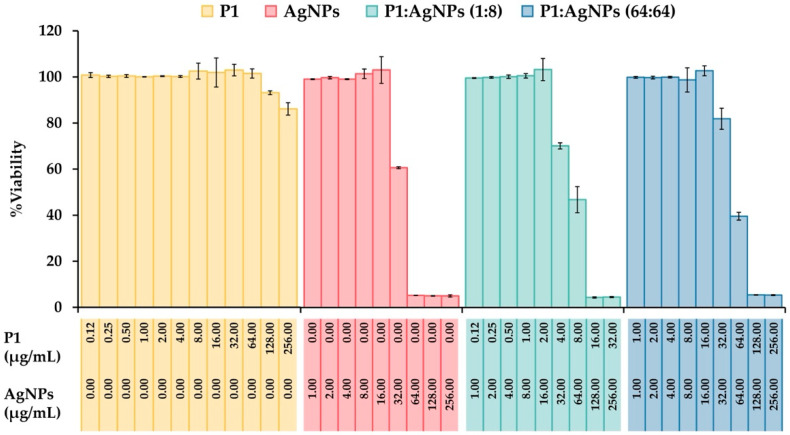
Cytotoxicity of the P1, AgNPs, and their combinations. Murine fibroblast L929 cells were treated with different concentrations of P1, AgNPs, and their combinations (1:8 and 64:64). The cell viability was assessed by MTT assay. PBS without samples was used as an untreated sample. The concentrations of P1 and AgNPs in the figure represent the final concentrations of each substance included in the mixture.

The cytotoxic effect of P1, AgNPs, and their combinations were evaluated using a 3-(4,5-dimethylthiazol-2-yl)-2,5-diphenyltetrazolium bromide (MTT) assay on the murine fibroblast L929 cell line. The results showed that P1 at concentrations up to 128 µg/mL provided cell viability of more than 90% (93.14 ± 0.79%) (Figure 10). AgNPs induced significant cytotoxicity at 32 µg/mL with reduced cell viability of 60.67 ± 0.42%. The SI values of P1 and AgNPs were 64 and 0.0625, implying that the AgNPs exhibited greater toxicity to the cell line than P1. This observation was consistent with previous studies, presenting that cationic AMPs and AgNPs exhibited dose-dependent cytotoxicity in mammalian cells. For example, synthetic WG18 peptide analogs showed cytotoxicity against RAW264.7 (murine macrophage) and HepG2 (human hepatocyte) cells in a dose-dependent manner. The optimal cationic charges on AMPs provided superior antimicrobial activity, but the highly positively charged peptides reduced cell viability [55]. AgNPs also showed cell toxicity that depended on their concentration, size, and exposure time. Small AgNPs (10–20 nm in size) at concentrations above 5 µg/mL readily penetrated the cells and stimulated ROS production, leading to mitochondrial damage and increased apoptotic cell death [56]. Moreover, the P1 and AgNP combinations (1:8 and 64:64) significantly reduced cell viability to 70.16 ± 1.34% and 81.93 ± 1.68% at concentrations of 4:32 and 32:32 µg/mL, respectively. The corresponding SI values were 2 and 0.25, supporting that a 1:8 combination was more selective to mammalian cells than a 64:64 mixture. The increased toxicity may be attributed to membrane disruption due to the actions of P1 and AgNPs. AgNPs-induced ROS also contributed to mammalian cell death. Careful optimization of the combined concentrations is necessary to balance antimicrobial efficacy with host cell safety.

### 2.11. Wound Healing Activity

**Figure 11:**
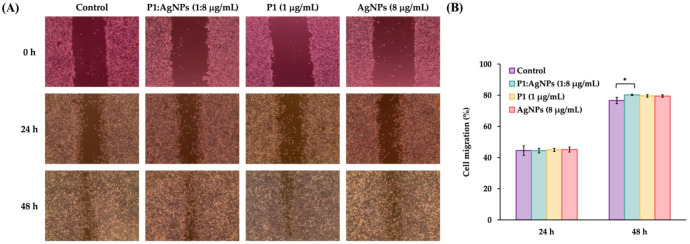
Cell migration in response to P1, AgNPs, and their combinations. (**A**) The samples (P1, AgNPs, and the 1:8 mixture) were incubated with the scratched fibroblast L929 cells for 24 and 48 h. (**B**) The wound width was measured, and the cell migration was calculated. An asterisk (*) indicates a significant difference at *p*-value < 0.05 compared to the untreated sample at each time point. The concentrations of P1 and AgNPs in the figure represent the final concentrations of each substance included in the mixture.

AgNPs have been demonstrated to heal wound injury by reducing the production of inflammatory cytokines and preventing bacterial infection. They also promote angiogenesis and increase collagen deposition, leading to the acceleration of wound healing [57,58,59]. To evaluate the ability of P1 and AgNP combinations on wound healing, the scratch-wound assay was performed using those combinations at concentrations that maintained cell viability above 90%. Therefore, only the 1:8 combination was selected for evaluation using the scratch-wound assay. It revealed that there are no significant differences in cell migration between treated and untreated samples at 24 h. The percent migrations of the 1:8 combinations were 44.48 ± 0.99% compared to that of the untreated sample (44.51 ± 3.04%) (Figure 11A). Cell migration following individual treatment with either P1 or AgNPs did not differ significantly from the treated sample, with percent migration values of 44.93 ± 1.62% or 45.18 ± 1.46%, respectively. However, the P1 and AgNP combination at a concentration of 1:8 μg/mL exhibited significantly higher migration (80.22 ± 0.82%) compared to the control (76.61 ± 2.05%) after 48 h (Figure 11B). Individual treatment with either P1 or AgNPs did not significantly promote cell migration compared to the untreated sample, with the migration of 79.58 ± 0.79% or 79.47 ± 0.42%, respectively. Our previous study demonstrated that P1 alone at a concentration of 200 µg/mL enhanced the migration of L929 fibroblast cells. The percentage of cell migration was 18.75 ± 1.44%, 60.33 ± 0.82%, and 100.00 ± 0.00% at 24, 48, and 72 h, respectively [60]. Moreover, a previous study reported that AgNPs suppressed fibroblast function by downregulating integrin-type receptors that bind to collagen and laminin. It resulted in loss of cell polarization, which impaired cell migration and possibly slowed the healing process [61]. On the other hand, the β-defensin-3 peptide could stimulate fibroblast migration and proliferation and promote angiogenesis, thereby supporting wound healing [62]. Therefore, these results suggest that, in addition to their antimicrobial properties, the P1 and AgNP combinations can promote cell migration, potentially indicating that the 1:8 combinations did not impede the wound healing process.

Thus, the P1 peptide combined with AgNPs showed an antimicrobial synergistic effect. The 1:8 mixture was identified as the most favorable combination, exhibiting antimicrobial efficacy against Gram-positive bacteria, low cytotoxicity, and high pro-migratory activity. The 64:64 co-treatment showed broad-spectrum activity against Gram-positive and Gram-negative bacteria and increased cytotoxicity. The low amount of the 64:64 mixture (0.25× MIC) displayed high cell viability, highlighting the importance of dose optimization for practical use. The P1 and AgNP combinations might be promising candidates for future development in the treatment of infection. However, there are some limitations in the study that require further investigation. It is necessary to evaluate the synergistic activity of the P1 peptide with more stable and less polydisperse AgNPs to consider that these factors considerably influence the observed antimicrobial activity. In-depth studies focusing on membrane permeability, Ag^+^ release, ROS production, alteration of intracellular molecules, DNA damage, and anti-inflammatory effects, will be useful to elucidate the exact mechanisms of the P1 and AgNP combinations on microbial eradication and wound healing.

## 3. Materials and Methods

### 3.1. Materials

Ammonium acetate, ammonium sulfate, silver nitrate (AgNO_3_), sodium chloride (NaCl), and trisodium citrate (C_6_H_5_O_7_Na_3_) were purchased from RCI Labscan Ltd., Bangkok, Thailand. Luria–Bertani (LB) broth and Mueller–Hinton (MH) agar were supplied by Titan Biotech Ltd., Rajasthan, India. Ampicillin and vancomycin were obtained from Sigma Aldrich Inc., St. Louis, MO, USA. Agarose, calcium chloride, ethylenediaminetetraacetic acid (EDTA), and magnesium sulfate were procured from Bio Basic Inc., Markham, ON, Canada. Acetonitrile (ACN), methanol, and trifluoroacetic acid (TFA) were obtained from Merck KGaA, Darmstadt, Germany. *S. aureus* TISTR 517 and *E. coli* TISTR 887 were from the Thailand Institute of Scientific and Technology Research (TISTR), Pathum Thani, Thailand. MRSA strain 2468 was provided by the Medical Technology Laboratory, School of Allied Health Sciences, Walailak University, Nakhon Si Thammarat, Thailand.

### 3.2. Chemical Synthesis of AgNPs

AgNPs were prepared by a chemical reaction between AgNO_3_ and C_6_H_5_O_7_Na_3_. A solution of 0.1 mM AgNO_3_ (50 mL) was heated, and a 1% trisodium citrate solution (5 mL) was gradually added. The mixture was continuously stirred and heated until it developed a pale yellow color, indicating the formation of AgNPs. It was allowed to cool to room temperature [63]. Subsequently, AgNPs were dialyzed by placing the sample solution into a membrane tubing with a cut-off value of 3500 Da (Thermo Fisher Scientific, Rockford, IL, USA) and immersing it in deionized (DI) water for 24 h. All procedures were carried out under light-protective conditions. The resulting colloidal AgNPs were stored at 4 °C for future experiments [64]. In addition, the AgNPs were completely dried using a freeze dryer (Martin Christ Gefriertrocknungsanlagen GmbH, Osterode am Harz, Germany) and weighed to determine their content for subsequent analysis.

### 3.3. Characterization of AgNPs and Their Mixtures with P1

#### 3.3.1. UV–Visible (UV–Vis) Spectroscopy

The physical appearance (color and uniformity) of AgNPs at a concentration of 100 µg/mL was evaluated. The absorbance peak of the colloidal AgNPs was recorded at the wavelength between 200 and 800 nm [64]. The spectrum was recorded using a V-630 spectrophotometer (JASCO Corporation, Tokyo, Japan) by setting a scanning speed at 1000 nm/min, a resolution of 1 nm, and a bandwidth of 1.5 nm. Baseline correction was achieved by using DI water as a blank. To characterize the interaction between P1 and AgNPs, the absorbance spectra of P1, AgNPs, and their mixtures (100 µg/mL) were measured using the same procedure.

#### 3.3.2. Particle Size and Zeta Potential

AgNPs (100 µg/mL) were dispersed in DI water and transferred into a quartz cuvette. A Nano-ZS ZEN 3600 Zetasizer (Malvern Instruments Ltd., Worcestershire, UK) was used to determine the hydrodynamic size of AgNPs by measuring dynamic light scattering at an angle of 90 degrees at 25 °C. The AgNPs were also loaded in a folded capillary cell, and the zeta potential was determined by calculating the electrophoretic mobility of the charged colloidal particles [65]. The same experimental procedure was applied to evaluate P1, AgNPs, and their mixtures at a concentration of 100 µg/mL.

#### 3.3.3. Fourier Transform-Infrared Spectroscopy (FT–IR)

The dried powders of P1, AgNPs, and their combinations were mixed with potassium bromide (KBr), compressed into pellets, and analyzed using an FT–IR spectrometer (Bruker Optik GmbH, Bremen, Germany) over a wavenumber range of 4000 to 400 cm^−1^. The instrument was operated at a resolution of 4 cm^−1^ with 16 scans per sample [65]. The IR spectra were examined to identify characteristic absorption bands and determine the functional groups.

#### 3.3.4. Powder X-Ray Diffraction (PXRD)

The dried powder of AgNPs (1 mg) was investigated by a Rigaku SuperNova diffractometer with a HyPix3000 detector (Rigaku Oxford Diffraction, The Woodlands, TX, USA) using Cu Kα radiation (λ = 1.54184 Å) [66]. The AgNPs were immobilized on an amine-functionalized glass surface before drying overnight under a vacuum. The experiment was performed at 25 °C, and the diffraction patterns were collected over an angular range of 10 to 90 degrees. In addition, the diffraction patterns of P1 and their mixtures (1 mg) were analyzed using the same experimental procedure.

#### 3.3.5. Transmission Electron Microscopy (TEM)

The morphology and size of AgNPs were characterized by a high-resolution JEM 2010 transmission electron microscope (Japan Electron Optics Laboratory Co. Ltd., Tokyo, Japan) at an operational voltage of 200 kV. The AgNP samples were loaded onto a carbon-coated grid and dried at room temperature under a vacuum. The TEM micrographs were captured at different magnifications. The size distribution of AgNPs was obtained from a histogram by counting at least 130 particles from multiple TEM images [67].

### 3.4. Preparation of P1 Peptide

A single colony of *Brevibacillus* sp. SPR20 was resuspended in 0.9% NaCl. The turbidity of the cell suspension was adjusted at an optical density (OD) of 625 nm equivalent to 0.1. This inoculum (2%) was added to the LB broth, and the culture was incubated at 30 °C for 24 h at 150 rpm. The cell-free supernatant was obtained by centrifugation, and then the P1 peptide was purified by salt precipitation, ion-exchange chromatography, and reversed-phase chromatography according to a previous study [9]. P1 was lyophilized and kept at 4 °C before use.

### 3.5. Determination of MIC and MBC

The MIC and MBC values of P1, AgNPs, and their combinations were assessed by the CLSI broth microdilution assay [68]. *S. aureus* TISTR 517, MRSA isolate 2468, and *E*. *coli* TISTR 887 were used as indicator bacteria. All bacteria were streaked on MH agar and maintained at 37 °C for 24 h. The colonies of each strain were resuspended in 0.9% NaCl, and the cell suspension was diluted to achieve an OD of 0.1 at 625 nm. The inoculum of indicator bacteria was diluted 20-fold with cation-adjusted Muller–Hinton broth (CAMHB) to obtain a cell density of 5 × 10^6^ CFU/mL. The samples (P1, AgNPs, and their combinations) were prepared in DI water, and the sample volume of 50 µL was transferred to each well of 96-well microplates. The CAMHB medium (50 µL) was then mixed with the samples to achieve their final concentrations in a range of 0.5 and 256 µg/mL. An aliquot of the diluted indicator bacteria (10 µL) was added, and the microplates were incubated at 37 °C for 24 h. Vancomycin and CAMHB with bacterial inoculum were used as positive and negative controls, respectively. The MIC value is defined as the minimum concentration of the sample at which no visible bacterial growth occurs. Moreover, 100 µL of samples at or above the MIC value were spread on MH agar and kept at 37 °C for 24 h. The MBC value is the minimum concentration of the sample required to kill bacteria.

### 3.6. Evaluation of Synergistic Effects

A broth microdilution checkerboard assay was used to assess the inhibitory effect of the P1 and AgNP combinations at specific concentrations. The P1 peptide was prepared at concentrations between 0.12 and 64 µg/mL for Gram-positive bacteria (*S. aureus* TISTR 517 and MRSA isolate 2468) and 4 to 2048 µg/mL for Gram-negative bacteria (*E*. *coli* TISTR 887). Twenty-five µL of each concentration of P1 was loaded onto a 96-well plate, and an equal volume of different concentrations of AgNPs (32–2048 µg/mL) was mixed with P1. The CAMHB medium (50 µL) was added, followed by the introduction of bacterial inoculum. The plates were kept at 37 °C for 24 h in an incubator, and the MIC value of each combination was recorded. The FICI was determined using Equation (2):

(2) $$\mathrm{FICI}\;=\;\frac{\mathrm{MIC}\;\mathrm{of}\;P1\;\mathrm{in}\;\mathrm{combination}}{\mathrm{MIC}\;\mathrm{of}\;P1\;\mathrm{alone}}+\;\frac{\mathrm{MIC}\;\mathrm{of}\;\mathrm{AgNPs}\;\mathrm{in}\;\mathrm{combination}}{\mathrm{MIC}\;\mathrm{of}\;\mathrm{AgNPs}\;\mathrm{alone}}$$

The FICI values are defined as synergistic (FICI ≤ 0.5), additive (0.5 < FICI ≤ 1.0), indifferent (1.0 < FICI ≤ 4.0), and antagonistic (FICI > 4.0) [41].

### 3.7. Time–Kill Assay

The time–kill test was used to evaluate the effect of the P1 and AgNP combination on bacterial growth. The indicator bacteria were grown and dispersed in CAMHB to obtain a cell density of 5 × 10^6^ CFU/mL. Different concentrations of P1, AgNPs, and their combinations were prepared in DI water. The samples (50 µL) and bacterial suspensions (50 µL) were mixed in microplates and then incubated at 37 °C. The samples were collected every 3 h over a 24 h study period. They were diluted with CAMHB before spreading the mixture on MH agar. The plates were maintained at 37 °C for 24 h, and the colonies were enumerated. The CAMHB medium with or without tested bacteria was used as an untreated sample or blank, respectively [43].

### 3.8. Drug Resistance Assay

The induction of antibiotic resistance of *S. aureus* TISTR 517 and *E. coli* TISTR 887 was performed by incubating the tested bacteria with P1, AgNPs, and their combinations at a sub-lethal dose (0.5× MIC value). The surviving cells were collected and then cultured in MH broth at 37 °C with shaking at 150 rpm until they grew into the exponential phase. The resultant bacteria were repeatedly treated with each sample at the 0.5× MIC level for 30 passages. Each bacterial passage measured the MIC value of P1, AgNPs, and their combinations by broth microdilution assay. Standard antibiotics (vancomycin and ampicillin) were used as the controls. The development of drug resistance of bacteria was evaluated by a change in the MIC of each passage normalized to that of the initial generation [69].

### 3.9. TEM of Treated Bacteria

The cell pellets of *S. aureus* TISTR 517 and *E. coli* TISTR 887 were harvested by centrifugation of their overnight cultures at 5000× *g* for 10 min, and they were washed twice with phosphate-buffer saline (PBS) pH 7.4. The pellets were dispersed in PBS and incubated with the P1 and AgNP combinations for 1 h. The samples were collected by centrifuging them at 5000× *g* for 2 min and washed with PBS. The bacterial cells were immobilized with 2.5% glutaraldehyde and sequentially dehydrated in 30%, 50%, 70%, and 90% ethanol for 10 min in each step, followed by twice rinsing in 100% ethanol. The samples were passed through propylene oxide and infiltrated with an epoxy resin. The polymerization was carried out at 70 °C for 12 h. The ultrathin sections of bacterial samples were prepared by an ultramicrotome (RMC Boeckeler Inc., Tucson, AZ, USA) and then subjected to a JEM 2010 transmission electron microscope (JEOL Ltd., Tokyo, Japan) at 200 kV [40].

### 3.10. DNA Binding Assay

The genomic DNA of *S. aureus* TISTR 517 and *E. coli* TISTR 887 was isolated using the GF-1 bacterial DNA extraction kit (Vivantis Technologies Sdn. Bhd., Selangor, Malaysia). The overnight cell culture (1 mL) was centrifuged at 6000× *g* for 2 min, and the pellets were resuspended and then treated with lysozyme at 37 °C for 20 min. The digested cells were centrifuged, and the pellets were resuspended in a buffer containing RNase A and Proteinase K. The mixture was loaded into a GF-1 column and centrifuged at 10,000× *g* for 1 min. A washing buffer was added to the spin column, followed by column centrifugation. Fifty µL of sterile water was used to elute genomic DNA. The bacterial genomic DNA (50 ng) was incubated with different concentrations of P1, AgNPs, and their combinations at 37 °C for 1 h [64]. The untreated and treated DNA samples were loaded on a 1% agarose gel before staining the gel with a Safe-Green dye after electrophoresis. The DNA bands were visualized by a ChemiDoc XRS+ imaging system (Bio-Rad Ltd., Hercules, CA, USA), and the band shifts in the gel were detected and evaluated by Image Lab software (version 6.1.0).

### 3.11. Hemolytic Activity of P1 and AgNP Combinations

A blood sample (5 mL) was drawn from a healthy volunteer and placed into an EDTA tube. It was centrifuged at 500× *g* for 5 min, and the erythrocytes in the lower part were collected. The cells were washed with PBS, followed by a centrifugation at 500× *g* for 5 min. The washing step was performed twice before diluting the erythrocyte pellet in PBS (1:25). The diluted cell suspensions (100 µL) were transferred to microcentrifuge tubes. The stock sample solutions (100 µL) containing P1, AgNPs, and their combinations were subsequently added to the tubes, resulting in the final concentrations ranging from 0.12 to 256 µg/mL. The tubes were incubated at 37 °C for 1 h, and the samples were collected by centrifugation at 500× *g* for 5 min. One hundred µL of the supernatant was added to a 96-well plate, and the absorbance at 451 nm was recorded using a Multiskan GO microplate reader (Thermo Fisher Scientific Oy, Vantaa, Finland). Positive and negative controls were performed in the same procedure using 1% Triton X-100 and PBS, respectively. The hemolytic activity was determined using the following Equation (3)

(3) $$\mathrm{Hemolytic}\;\mathrm{activity}\;(\%)=\frac{A_{\mathrm{sample}}-A_{\mathrm{negative}}}{A_{\mathrm{positive}}-A_{\mathrm{negative}}}\times \;100$$

where A_sample_, A_positive_, and A_negative_ are the absorbance of the sample, positive, and negative controls, respectively [70].

### 3.12. In Vitro Cytotoxicity Evaluation

To assess the safety of the P1 and AgNP combinatory samples, a MTT assay was used to evaluate cell viability, according to a previous study [70]. Murine fibroblast L929 cell line (Chinese Academy of Preventive Medical Sciences, Beijing, China) was cultured in Dulbecco Modified Eagle Medium (DMEM) supplemented with 10% fetal bovine serum (FBS) and antibiotics (100 units/mL penicillin and streptomycin). The cells were maintained at 37 °C with a 5% CO_2_ level. When they reached confluence, they were collected using 0.25% trypsin-EDTA (Thermo Fisher Scientific Inc., Grand Island, NY, USA). The cells (1 × 10^5^ cells/mL) were seeded into a 96-well plate. After a 24 h incubation, the old medium was discarded, and the fresh medium (100 µL), comprising P1, AgNPs, and their combinations at different concentrations, was added. The medium containing phosphate-buffer saline was used as a negative control. The cells were incubated for 24 h, followed by a medium change with medium containing 3-(4, 5-dimethylthiazolyl-2-yl)-diphenyl-tetrazolium bromide (MTT). They were incubated at 37 °C under 5% CO_2_ for 4 h. Subsequently, the culture medium was removed, and 100 µL of dimethyl sulfoxide (DMSO) was added to solubilize the insoluble purple formazan product. The absorbance at 570 nm was measured by a Biohit 830 microplate reader (Biohit Oyj, Helsinki, Finland) [60]. The cell viability was calculated as a percentage compared to the negative control, as shown in Equation (4).

(4) $$\mathrm{Cell}\;\mathrm{viability}\;\left(\%\right)=\frac{A_{\mathrm{sample}}}{A_{\mathrm{negative}}}\text{×}\;100$$

where A_sample_ and A_negative_ are the absorbances of treated and untreated cells, respectively.

### 3.13. Scratch-Wound Assay

The scratch assay was employed to measure the cell migration, representing the wound-healing activity of the samples. The L929 fibroblast cells were plated in a 6-well plate at a density of 5 × 10^4^ cells per well and incubated until they formed a confluent layer. A scratch on the cell monolayer was made by a sterile pipette tip. The detached cells were eliminated by rinsing with PBS, and the DMEM (2 mL), containing P1 and AgNP combinations at their highest non-cytotoxic concentrations, was added. Individual treatments with either P1 or AgNPs at the corresponding concentrations were also conducted. The cells were incubated at 37 °C for 24 h in a 5% CO_2_ incubator. The untreated cells were used as the control. The cell migration was observed under a CK2 microscope (Olympus Optical Co. Ltd., Tokyo, Japan) at 10× magnification, and the images were taken at 0, 24, and 48 h. The distance between wound edges was analyzed by ImageJ (version 1.54p), and the percentage of cell migration was analyzed by the following Equation (5) [71].

(5) $$\mathrm{Migration}\;\mathrm{rate}\;\left(\%\right)=\frac{D_{i}-\;D_{f}}{D_{i}}\times \;100$$

where D_i_ and D_f_ are the distances between the edges of the scratch at the initial and final wound width, respectively [72].

### 3.14. Statistical Analysis

Each experiment was performed in three independent replicates. The result was reported as mean ± standard deviation (SD). Student’s *t*-test or one-way analysis of variance (ANOVA) was used to determine the statistical difference at a *p*-value of less than 0.05.

## 4. Conclusions

The P1 peptide showed antimicrobial efficacy against Gram-positive bacteria (*S. aureus* and MRSA). Its combination with AgNPs exhibited a synergistic effect. The P1 and AgNP mixture (1:8 µg/mL) showed twice the activity against Gram-positive bacteria. A higher concentration of the mixture (64:64 µg/mL) expanded its spectrum to include Gram-negative bacteria (*E. coli*) with a 32-fold increase in activity. These combinations exhibited bactericidal activity in a concentration-dependent manner, showing a high killing rate and completely eradicating bacteria within 3 h when tested at 10× MIC. The combined treatment of P1 and AgNPs induced little to no antimicrobial resistance compared to standard antibiotics or individual treatments with P1 or AgNPs. The synergistic action of the combinations involved the disruption of cell walls and membranes, leakage of the intracellular content, and interaction with bacterial genomic DNA, resulting in cell death. The P1 and AgNP mixture at a concentration of 1:8 µg/mL exhibited hemocompatibility and low cytotoxicity, whereas the 64:64 µg/mL mixture resulted in increased hemolysis and cell toxicity. Moreover, the 1:8 combination did not impede the wound healing process.

## Figures and Tables

**Table 1:** Hydrodynamic size, zeta potential, and PDI of AgNPs, P1, and mixtures of P1 and AgNPs.

Compound	Hydrodynamic Size (nm)	Zeta Potential (mV)	PDI
AgNPs	46.3 ± 0.5	−20.58 ± 0.69	0.578 ± 0.004
P1	439.4 ± 34.0	0.23 ± 0.22	0.348 ± 0.108
P1:AgNPs (1:8)	2275.3 ± 0.6	−11.39 ± 0.57	0.709 ± 0.050
P1:AgNPs (64:64)	2700.7 ± 1.1	−7.49 ± 0.52	0.667 ± 0.096

**Table 2:** The MIC, MBC, and FICI of P1, AgNPs, and their combinations against *S*. *aureus* TISTR 517, MRSA isolate 2468, and *E*. *coli* TISTR 887.

Tested Bacteria	Agent	MIC (µg/mL)	MBC (µg/mL)	P1 and AgNP Combination			
				MIC (µg/mL)	FICI	Fold Change	Interpretation
*S. aureus* TISTR 517	P1	2	4	1	0.5	2	Synergy
	AgNPs	256	256	8			
	Vancomycin	2	2	ND	ND	ND	ND
MRSA isolate 2468	P1	2	4	1	0.5	2	Synergy
	AgNPs	256	256	8			
	Vancomycin	2	4	ND	ND	ND	ND
*E. coli* TISTR 887	P1	256	256	64	0.5	32	Synergy
	AgNPs	256	256	64			
	P1	256	256	128	0.5	2	Synergy
	AgNPs	256	256	8			
	Ampicillin	4	8	ND	ND	ND	ND

ND denotes not determined.

## Data Availability

Data are available within the article and Supplementary Materials. Reasonable inquiries for additional information can be directed to the corresponding author.

## Supplementary Materials

The following supporting information can be downloaded at https://www.mdpi.com/article/10.3390/ijms26167832/s1.
