# Supplementary material for: Synergistic Antimicrobial Activity of BrSPR20-P1 Peptide and Silver Nanoparticles Against Pathogenic Bacteria
Source: Int J Mol Sci. 2025 Aug 13;26(16):7832. doi: 10.3390/ijms26167832 (PMC12386432; doi:10.3390/ijms26167832)
Supplement: Supplementary file 1 [file ijms-26-07832-s001.zip › ijms-3813819-supplementary.pdf]

Supplementary Materials: Synergistic Antimicrobial Activity of BrSPR20-P1 Peptide and Silver Nanoparticles Against Pathogenic Bacteria

Thanyamai Thongin [1], Somchai Sawatdee [1,2], Nuttapon Songnaka [1,2], Jumpei Uchiyama [3], Theanchai Wiwasuku [4], Teerapol Srichana [5], Titpawan Nakpheng [5] and Apichart Atipairin [1,2,*]

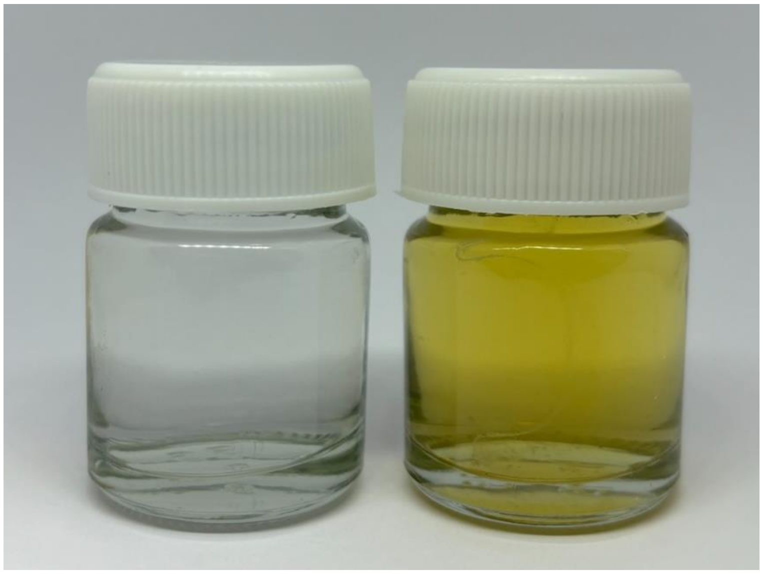

Figure S1. Visual appearance of $AgNO_3$ solution (left) and synthesized AgNPs (right).

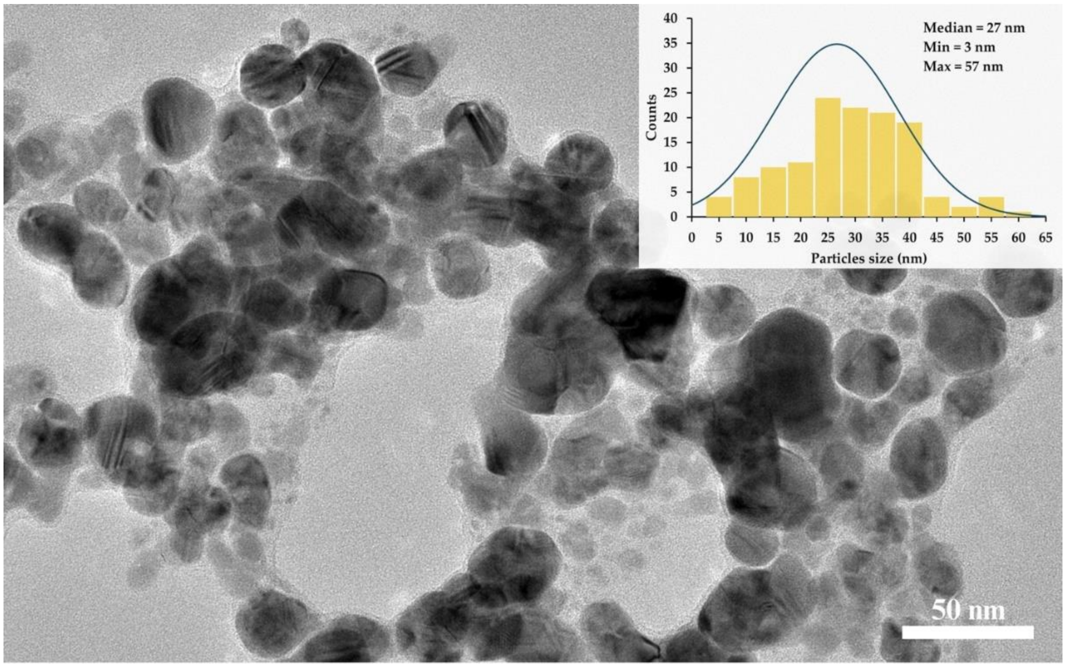

Figure S2. Morphology of AgNPs at 150,000× magnification, with the inset showing the particle size distribution derived from TEM micrographs.
